# Magnitude and associated factors of antenatal depression among mothers attending antenatal care in Arba Minch town, Ethiopia, 2018

**DOI:** 10.1371/journal.pone.0260691

**Published:** 2021-12-02

**Authors:** Eskedar Demissie Beketie, HaileMariam Berhe Kahsay, Fiseha Girma Nigussie, Wubishet Tesfaye Tafese

**Affiliations:** 1 Department of Nursing, College of Medicine and Health Science, Wolkite University, Wolkite, Ethiopia; 2 School of Nursing, College of Medicine and Health Science, Mekelle University, Mekelle, Ethiopia; 3 Department of Nursing, College of Medicine and Health Science, Debrebirhan University, Debirebirhan, Ethiopia; University of Mississippi Medical Center, UNITED STATES

## Abstract

**Background:**

Depression is a common mental disorder. The burden of antenatal depression is higher in developing countries which is 20% as compared to developed ones 10% to 15%. In Ethiopia around one-fifth of pregnant mothers are depressed. Despite the severity of the problem, only a few studies have been done in Ethiopia, and there is no study done in Arba Minch on the problem.

**Objective:**

To assess the magnitude and associated factors of antenatal depressive symptoms among pregnant women attending Public Health facilities in Arba Minch town Southern Nations and Nationalities Peoples Region, Ethiopia 2018.

**Methods:**

Health Institution based, cross-sectional study design was used to assess the magnitude and associated factors of antenatal depression among 323 pregnant mothers who came for antenatal care follow-up in all public health facilities in Arba Minch town. The systematic random sampling technique was applied. Interviewer administered, pretested structured Questionnaire containing Edinburgh postpartum depression scale was utilized. EPI INFO was used to enter data and then the data were analyzed by logistic regression using SPSS. Variables with P-value less than 0.2 in the bivariate logistic regression were inserted in for multivariable analysis to see their independent effect and those with P-value less than 0.05 were used to determine the significant association between dependent and independent variables.

**Result:**

The magnitude of antenatal depression was 35.4%. Variables that were significantly associated with antenatal depression on multivariate analysis were anxiety (AOR = 5.49, 95%CI: 2.56, 11.77), un-planned pregnancy (AOR = 2.71, 95%CI: 1.21, 6.07), and Primigravida (AOR = 2.96, 95%CI: 1.28, 6.8). Similarly, uneducated mothers and those who attend only elementary school had AOR 4.92, 95% CI 1.36,17.73 and AOR 4.04955CI 1.23, 13.39 respectively.

**Conclusion:**

The magnitude of antenatal depression, intimate partner violence, and threatening life event in Arba Minch town was high. Anxiety, unplanned pregnancy, educational status, and Primigravida were significantly associated factors with depression. There should be a mechanism for routine screening and management of antenatal depression and intimate partner violence during antenatal care follow-up.

## Introduction

Mood is a pervasive and sustained emotion or feeling tone that influences a person’s behavior and colors his or her perception of being in the world [1]. Depression is one of the mood disorders in which World Health Organization (WHO) defines it as a common mental disorder which presents with a profound and persistent feeling of sadness, disturbed sleep or appetite, loss of interest or pleasure, feelings of guilt, or low self-worth, decreased energy, and poor attention [2].

Despite the presence of effective treatment, depression is one of the leading causes of disease burden in the world [3]. WHO’s report in 2015 showed that the prevalence of depression in the general population was 4.4% which is higher among females (5.1%) as compared with males (3.6%) [4]. Throughout the world, around 10% of mothers experience mental problems during their pregnancy and its burden is higher in low and middle-income countries which is around 15.9% and depression is one of the main mental disorders occur during pregnancy [5, 6]. The burden of antenatal depression is higher in developing countries which is 20% as compared to developed ones 10% to 15% [7]. In Ethiopia more than one-fifth (23.56%) of pregnant mothers are depressed [8].

Antenatal depression could result in short and long-term adverse outcomes for both the baby and the mother. Its effect ranges from the limitation of everyday activity and participation restrictions to poor dietary intake. About half of the mothers with antenatal depression will develop postpartum depression and has suicide ideation [9–13]. Antenatal depression also affects the well-being of the baby from its development in the uterus to their adulthood. Some studies showed that babies of depressed mothers have low birth weight and preterm birth. It could also lead to fetal growth retardation, low APGAR score, and small for gestational age babies [12, 14, 15]. Similarly, children born from mothers who had antenatal depression develop, depressive disorder and commit violent acts during their adolescence [16, 17].

Most studies revealed that unwanted pregnancy, poor social support, low economic status, previous history of psychiatric illness, abortion, and stillbirth are associated with depression, and some studies have contradicting results on the association between these factors and antenatal depression [18–20]. Edinburgh postpartum depression scale (EPDS) is validated and approved for screening antepartum and postpartum depression in both urban and rural Ethiopia. It has been used to assess antepartum depression in researches but there is no national strategy for routine screening of mothers on ANC follow up [21, 22].

Despite the severity of the problem, only a few studies have been done in Ethiopia. Almost all the studies failed to study the association of antenatal depression with anxiety. In addition, there is no study done in Arba Minch town on the problem.

Since this study included anxiety, which was neglected in previous studies, but could have a significant association with the problem, the finding of the study could be helpful in improving knowledge on factors associated with the problem for maternal health disciplines. It will also be helpful to design and implement maternal health policies and programs for policymakers in Ethiopia. Since there are only a few studies done on the topic in Ethiopia, this study will be used as a baseline for other studies.

## Material and methods

### Study setting, design and participants

The study was conducted in Arba Minch town, a capital of the Gamo Gofa zone, southern Ethiopia. It is 455 km away from Addis Ababa, the capital city of Ethiopia. The town has an estimated total population of 159,019 in 2018 of which 79,058 are females and 79,961 are males. Reproductive age group women are 37, 052. The town has one hospital and two health centers, which provide ANC service. The study was conducted from September 1 to October 31, 2018, on both health centers and the hospital.

Health Institution based cross-sectional study design was used to conduct the study. All pregnant women attending Public Health facilities for antenatal care in Arba Minch town were the source population and those who were attending ANC during the study period were the study population. A sample size of 323 was found by using single population proportion formula taking 5% Margin of error, 95% CI, and estimated magnitude of antenatal depression 25.6% [23]. Calculating the average of the previous three month ANC flow of each health institution the sample size was proportionally allocated. Then systematic random sampling technique was implemented to select study participants (the data was collected from every four mothers who came for ANC visits in each health institution.)

Three BSC midwives and one Msc nurse were recruited as data collectors and supervisor, respectively. Then they took two days of training on interviewing techniques, the objective of the study, and different sections of the questionnaire.

### Sampling technique and procedure

The research was conducted at two health centers (Arba Minch and Shecha health centers) and Arba Minch referral hospital. The sample size split between health institutions is proportional to their ANC caseload. Calculating the average of the previous three-month ANC flow, the monthly caseload of Arba Minch referral hospital, Arba Minch health center, and Shecha health centers were 595, 507 and 158 respectively. The sample size was proportionally allocated, and 153 samples were taken from Arba Minch referral hospital; 130 and 40 samples were taken from Arba Minch and Shecha health centers, respectively. Then systematic random sampling technique was implemented to select study participants.

### Eligibility criteria

All pregnant women, irrespective of their trimester, who came for ANC follow-up in public health facilities of Arba Minch town during the data collection period, were included in the study. Mothers who were unable to listen or speak and those with serious medical conditions were excluded.

### Data collection method and instrument

Data were collected using an interviewer-administered, pretested structured questionnaire containing Edinburgh Postnatal Depression Scale (EPDS), which was used to assess antepartum depression. EPDS has ten questions; each scored zero to three, the total score ranging from zero to 30. As the total score gets higher, the mother has higher depressive symptoms. It was validated to detect antepartum and postpartum depression in many countries [24–26]. It also showed a sensitivity of 84.6% and a specificity of 77.0% in Addis Ababa for postpartum use. It had Cronbach’s Alpha of 0.71, and areas under receiver operating characteristics were 0.85 [21]. Like previous studies done in Ethiopia, mothers who scored ≥ 13 and < 13 on EPDS were considered as positive, and negative for antepartum depression screening, respectively [27].

Generalized Anxiety Disorder (GAD) 7-Item Scale was used to assess GAD. It was used in the study conducted in Ethiopia and had Cronbach’s Alpha of 0.917 [28]. Mothers who scored < 7 were grouped as having no GAD, and those with a score ≥ 7 were grouped as having GAD [29]. Maternity social support score (MSSS), which contains six items, was used to assess social support. Mothers with maternity social support scores of 24–30 were grouped as high social support; 18–23 medium social support, and low social support were those below 18 [30]. Abuse Assessment Screen (AAS), which contains five items was used to assess intimate partner violence, and studies showed that it has a sensitivity of 93% -94% and a specificity of 55% -99% [31–33]. Mothers who responded to any question on the abuse assessment screen affirmatively considered positive for abuse [34]. Twelve lists of threatening life events were used to assess life stressors. The TLE has good test-retest (Kappa: 0.61–0.87) and predictive validity [35]. Mothers who experienced at least one stressful life event in the past six months were grouped as yes for threatening life events, and those without any stressful life events were grouped as no for threatening life events [18]. All the tools used in this study were used in other studies done in Ethiopia.

Questions that assess other variables like socio-demographic factors, obstetric and gynecologic factors, and Substance use were adopted from literatures. Medical records were reviewed to observe gestational age and the presence of complications in the current pregnancy. History of depression on the mother and her family was assessed by asking the mother whether she or her family had a known history of depression, which was diagnosed by a health professional. Mothers also asked about the feeling of their husband (partner) on the pregnancy, whether he was happy or unhappy about the pregnancy, and supports the mother during her pregnancy.

### Data analysis

EPI INFO version 3.5.1 statistical software was used to enter the data, then SPSS version 20 statistical package was used for analysis. The Hosmer-Lemeshow method was used to assess model fitness, and it was found to be a well-fit model. Multi-collinearity was checked for the three most related variables (Gravidity, parity, and the number of babies the mother had in her home) and found to have a Variance Inflation Factor (VIF) value greater than 10, indicating there is multi-collinearity among these variables. Thus, gravidity was used for analysis. Since generalized anxiety and depression share some symptoms, they are checked for collinearity, and VIF was one indicating there is no collinearity between these two variables.

Binary logistic regression with 95% CI was used to explore the relationship between antenatal depression and other independent variables. First bivariate logistic regression was used, and those variables with P-value less than 0.2 were inserted into multivariable analysis to see their independent effect. Variables with P-value less than 0.05 were used to determine a significant association between dependent and independent variables. The variables which do not include values of primigravid or primipara mothers are excluded on multivariable analysis.

### Ethical considerations

The study was approved by the Mekelle university college of health science ethical review board. An official letter was written to Gamo Gofa Zone Health Department to get permission for data collection. Informed written consent was obtained from the respondents after explaining the purpose of the data collection and Privacy of respondents in the study by no means infringed. Response of the participant recorded and analogized anonymously. Moreover, respondents were given the freedom to refuse their participation at any stage of this study.

## Results

### Socio demographic characteristics

A total of 316 mothers participate in the study giving a response rate of 97.8%. the mean age of mothers was 25.8 (±5.4 SD) with a minimum of 15 and a maximum of 40 years old, and more than half of them (60.1%) were between 20 and 29 years old. The monthly income of 22.6% of respondents was less than or equal to 1000 Birr. (Table 1)

**Table 1:** Socio demographic characteristics of pregnant women attending public health facilities in Arba Minch SNNPR Ethiopia 2018.

Variables (n = 316)	Categories	Frequencies	Prevalence (%)
**Ethnicity**	Gamo	222	70.3
	Wolayta	45	14.2
	Amhara	31	9.8
	Others	18	5.7
**Religion**	Orthodox	168	53.2
	Protestant	126	39.9
	Others	22	6.9
**Age**	< 20	39	12.3
	20–29	190	60.1
	≥ 30	87	27.5
**Monthly income (n = 296)**	≤ 500 birr	30	10.1
	501–1000 birr	37	12.5
	≥1001	229	77.4
**Marital status**	Married	292	92.4
	Not married	24	7.6
**Education**	Non-educated	65	20.6
	Educated	251	79.4
**Educational status**	Non-educated	65	20.6
	Elementary school	73	23.1
	Secondary school	81	25.6
	Above secondary school	97	30.7
**Occupation**	Government employee	62	19.6
	Private employee	22	7
	Running personal business	35	11.1
	House wife	115	36.4
	Student	51	16.1
	Jobless	31	9.8

Ethnicity others- Konso, Gurage, Oromo, Tsema, Tigre

Religion others- Jehovah’s Witness.

### Magnitude of antenatal depression

Among 316 respondents, 201 (64.6%) had EPDS score < 13, and the rest 110 (35.4%) had EPDS scores greater or equal to 13, making the overall magnitude of antenatal depressive symptoms among study participants 35.4%.

### Obstetric and gynecologic factors

More than half of respondents 205 (64.9%) had a regular menstrual cycle, and 183 (57.9%) used contraceptives before the current pregnancy. About 76.3% of the respondent planned their pregnancy while the rest 23.7% had unplanned (unintended) pregnancy. The current pregnancy was the first pregnancy for 149 mothers. Among those who were multigravida, about 86.8% had ANC follow-up for the previous pregnancies. About 37 (22.2%) of multigravida mothers had complications in their past pregnancy and of these 40.5% had preterm labor. About 258 (81.6%) respondents had no complications on the current pregnancy. (Table 2)

**Table 2:** Obstetric factors among pregnant women attending public health facilities in Arba Minch SNNPR Ethiopia 2018.

Variables	Categories	Frequencies	Prevalence (%)
Pattern of menstrual cycle before the pregnancy	Regular	205	64.9
	Irregular	111	35.1
Contraceptive utilization	Yes	183	57.9
	No	133	42.1
Type of contraceptive (n = 183)			
	OCP	12	6.6
	Injectable	120	65.6
	Implant	42	22.9
	IUCD	9	4.9
Pregnancy intention	Intended	241	76.3
	Un-intended	75	23.7
Gravidity	Primigravida	149	47.2
	Multigravida	167	52.8
Previous ANC follow up (n = 167)	Yes	145	86.8
	No	22	13.2
Way of previous ANC follow up (n = 145)	Regularly	139	95.8
	Irregularly	6	4.1
Complication in the past pregnancies (n = 167)	Present	37	22.2
	Absent	130	77.8
Type of complications in the past pregnancies (n = 37)	Abortion	11	29.7
	Preterm labor	15	40.5
	Stillbirth	5	13.5
	Others	6	16.2
Gestational age	First trimester	24	7.6
	Second trimester	145	45.9
	Third trimester	147	46.5
Complication in the current pregnancy	Present	58	18.4
	Absent	258	81.6

Type of complication others–hypertension, hyperemesis gravidarum and anemia.

### Psychosocial factors

More than half of mothers 195 (61.7%) experienced at least one threatening life event in the past six months before data collection, and about 54.1% had high social support. About 20.9% of respondents had intimate partner violence. About 281 (88.9%) of the husbands (partners) were happy on the pregnancy, and about 93.4% of them provided good support for their wives (partners). (Table 3)

**Table 3:** Psychosocial factors among pregnant women attending public health facilities in Arba Minch SNNPR Ethiopia 2018.

Variables (n = 316)	Categories	Frequencies	Valid percent (%)
Threatening life event (TLE)	Absent	121	38.3
	Present	195	61.7
Social support (MSSS)	High social support	171	54.1
	Medium social support	114	36.1
	low social support	31	9.8
Intimate partner violence (AAS)	Absent	250	79.1
	Present	66	20.9
Baby’s father feeling in the pregnancy	Happy	281	88.9
	Un-happy	35	11.1
Baby’s father support	Poor	20	6.3
	Good	296	93.7

### Mental health conditions

About 10.8% of mothers had a family history of depression, and 19.6% of respondents had a personal history of depression. Similarly, more than one-fifth (23.1%) of respondents had a score of seven and above on the GAD seven item scale indicating positive for anxiety screening.

### Substance use

The majority of respondents didn’t smoke a cigarette or chew khat during their pregnancy. About 17.1% of them drunk alcohol at least once during their pregnancy. (Table 4)

**Table 4:** Substance use among pregnant women attending public health facilities in Arba Minch SNNPR Ethiopia 2018.

Variables (n = 316)	Categories	Frequencies	Valid percent (%)
Drunk alcohol at least once in the pregnancy	Yes	54	17.1
	No	262	82.9
Chew khat at least once in the pregnancy	Yes	2	0.6
	No	314	99.4
Smoke cigarette at least once in the pregnancy	Yes	18	5.7
	No	298	94.3

### Associated factors

In the bivariate analysis the variables which had a significant association (P-Value < 0.2) with antenatal depression were age, income, educational status, occupation, marital status, gravidity, social support, threatening life events, intimate partner violence, husband (partner) feeling on the pregnancy, husband (partner) support, current complication on the pregnancy, pregnancy intention and anxiety.

In multivariable logistic regression only anxiety, pregnancy intention, educational status, and gravidity were significantly associated. The odds of developing antenatal depression were more than five times higher among mothers having anxiety as compared with those who have no anxiety(AOR = 5.49, 95%CI: 2.56, 11.77). The odd of antenatal depression was about three times higher among mothers who didn’t plan to conceive and primigravids (AOR = 2.71, 95%CI: 1.21, 6.07) and (AOR = 2.96, 95%CI: 1.28, 6.81) respectively. Similarly, mothers who had antenatal depression were about five and four times more likely to be uneducated and attend only elementary school than mothers who attended above secondary school, respectively. (Table 5)

**Table 5:** Factors associated with depression among pregnant women attending public health facilities in Arba Minch SNNPR Ethiopia 2018.

Variables	Antenatal depression		COR(95% C.I OR)	AOR (95% C.I)
	Yes(n)	No(n)		
**Age**				
** <20**	22	17	4.96(2.19,11.25)	0.99(0.25,3.94)
** 20–29**	72	118	2.34(1.29,4.24)	1.13(0.45,2.85)
** ≥30**	18	69	1.00	1.00
**Income**				
** ≤** **500 birr**	22	8	7.40(3.13,17.50)	1.94(0.45,8.35)
** 501–1000 birr**	20	17	3.17(1.56,6.44)	2.39(0.95,6.00)
** ** **≥** **1001**	62	167	1.00	1.00
**Educational status** *				
** Non-educated**	22	43	2.25 (1.08,4.64)	4.92(1.36,17.73)
** Elementary school**	39	34	5.03(2.53,10.01)	4.04(1.23,13.39)
** Secondary school**	33	48	3.01(1.53,5.94)	2.68(0.95,7.51)
** Above secondary school**	18	79	1.00	1.00
**Occupation**				
** Private employee**	9	13	3.6(1.21,10.67)	0.61(0.10,3.52)
** Running personal business**	8	27	1.54(0.55,4.36)	0.46(0.11,2.00)
** House wife**	42	73	2.99(1.38,6.50)	0.76(0.22,2.67)
** Student**	24	27	4.62(1.93,11.05)	1.49(0.44,5.11)
** Jobless**	19	12	8.23(3.06,22.16)	1.23(0.29,5.24)
** Government employee**	10	52	1.00	1.00
**marital status**				
** Married**	96	196	4.08(1.69,9.88)	0.86(0.18,4.23)
** Not married**	16	8	1.00	1.00
**Gravidity** *				
** Primigravida**	69	80	2.49 (1.55, 3.99)	2.96(1.28,6.81)
** Multigravida**	43	124	1.00	1.00
**Social support**				
** low social support**	23	8	7.16(2.99,17.09)	1.95(0.56,6.80)
** Medium social support**	40	74	1.35(0.81,2.24)	0.64(0.32,1.29)
** High social support**	49	122	1.00	1.00
**Threatening life events**				
** Present**	80	115	1.94(1.183.17)	1.56(0.78,3.13)
** Absent**	32	89	1.00	1.00
**Intimate partner violence**				
** Present**	36	30	2.75(1.58,4.78)	1.11(0.49,2.51)
** Absent**	76	174	1.00	1.00
**Husband (partner) feeling on the pregnancy**				
** Happy**	88	193	4.79(2.25,10.20)	2.44(0.73,8.18)
** Un-happy**	24	11	1.00	1.00
**Husband (partner) support**				
** Poor**	14	16	4.71(1.76,12.64)	0.31(0.47,2.06)
** Good**	98	198	1.00	1.00
**Complication in current pregnancy**				
** Present**	30	28	2.30 (1.29, 4.11)	2.06(0.92,4.61)
** Absent**	82	176	1.00	1.00
**Pregnancy intention** *				
** Un- planned pregnancy**	47	28	4.55 (2.63, 7.86)	2.71(1.21,6.07)
** Planned pregnancy**	65	176	1.00	1.00
**Anxiety** *				
** Present**	51	22	6.92 (3.88, 12.33)	5.49(2.56,11.77)
** Absent**	61	182	1.00	1.00

* = Significantly associated.

## Discussion

This study investigated the magnitude and associated factors of antenatal depressive symptoms among pregnant mothers attending Public Health facilities for antenatal care in Arba Minch town. The magnitude of antenatal depressive symptoms in this study was 35.4% (95% CI: 30–41). It was higher as compared with a systematic review done in developed and developing countries, which were about one-tenth in developed and one-fifth in developing countries [7]. Some studies done in developing countries like India, Tanzania, and Cape Town had a consistent prevalence of antenatal depression with the current study which ware 36.75%, 33.8%, and 39% respectively [19, 36, 37]. Studies done in Ethiopia had a lower magnitude of antenatal depression, which ranges between 11.8%– 25.6%, as compared with the current study [18, 23, 27, 30, 38]. This could be due to the difference in the trimester of the mother they used in which the study done in south-west Ethiopia exclude those in the first trimester and the tool used by the study done in Gondar was BDI which was different from EPDS. On the other hand, mothers with significantly associated factors like un-intended pregnancy and being primigravida were higher in this study as compared with the previously stated studies. These could have contributed to a higher magnitude of antenatal depression in this study.

In this study, the prevalence of IPV is about 21%. Among these, about 12% of them were afraid of their partner, and 9.5% had been hit, slapped, kicked, or otherwise physically hurt by their partner. Most rural communities of Ethiopia condone IPV that wives should be afraid of their husbands (partners) and it is their right to beat them [39]. This result is relatively lower as compared with the systemic review done in Ethiopia, which was 26% [40]. On the other hand, the prevalence of IPV is higher than the study done in southwestern Ethiopia. This could be due to the difference in the tool used in this study included both physical and emotional abuse. On the other hand, the study conducted in south-west Ethiopia included only the physical abuse [30].

The prevalence of threatening life events is about 62%, of which about 20% of mothers lost their close family, friend, or second-degree relative by death, and bout 15% of the mothers were unemployed or seek work for more than one month. This result is higher than the study done in South Africa, which was 39%. This could be due to the score we used in this study was mothers who had at least one TLE are grouped as having TLE. Whereas, the study done in South Africa used at least two TLE to say a mother had a stressful life event [11].

Complications in the past pregnancies had no association with antenatal depression in this study. Similarly, the study done in Cape Town showed there was no association between complications in previous pregnancies and antenatal depression [19]. On the contrary, studies done in Malawi, China, and Debretabor indicated that complications in the previous pregnancies like miscarriage and stillbirth had a significant association with the outcome variable [18, 20, 41]. This could be due to the difference in the magnitude of complications in the past pregnancies, in which this study had a lower magnitude as compared with the study done in Debretabor. The difference in the tools they used in the studies done in China and Malawi also might attribute to the difference in the findings.

Though most studies showed that social support and intimate partner violence had an association with antenatal depression, in this study both predictors had no association with depression [11, 30, 42]. This could be due to the tool they used to assess IPV and social support was different. Unlike the studies done in South Africa and Debretabor, stressful life events in the past six months were not significantly associated with antenatal depression in this study [11, 18]. Though the variables were not significantly associated with the outcome variable in multivariate analysis majority of mothers in this study had good social support, a happy partner (husband) about the pregnancy, and good support from him, which might help her out to withstand threatening life events that she faced in the past six months.

In this study, the husband’s (partner’s) feeling on the pregnancy was not significantly associated with antenatal depression. Similarly, the study done in Addis Ababa revealed a similar result [27]. The finding in this study also showed that there was no association between husband (partner) support and antenatal depression. On the contrary, the study done in Addis Ababa showed that mothers without the baby’s father’s support were more than twice as likely to develop antenatal depression as compared with women who got support from the baby’s father [27]. The difference in social interaction among the community between the two areas might attribute to the difference in the finding.

This study had similar finding regarding the previous history of depression with the study done in South Africa in which there is no association with the variable and antenatal depression. On the contrary, the studies were done in Tanzania, Debretabor, and Addis Ababa indicated there was a significant positive association between history of depression and antenatal depression. Family history of depression also has no association with antenatal depression in this study, which is consistent with the study done in Addis Ababa [18, 27, 37]. Lack of awareness on mental health problems and poor trend of the community to seek treatment for mental health problems might attribute to the difference in findings.

This study showed that mothers who were not educated, and those who attend only elementary school had about five and four times higher odds of developing antenatal depression as compared with mothers who were educated above secondary school respectively. onthe other hand, cross-sectional studies done in Cape Town and Addis Ababa indicated that there was no association between the educational status of the mother and antenatal depression [19, 27].

Mothers who didn’t plan their pregnancy had 2.71 times higher odds of developing antenatal depression as compared with those who planned their pregnancy. This result is in line with the study done in Oman in which unplanned pregnancy had a significant positive association with antenatal depression with an OR of 1.37 [43]. Similarly, studies done in Addis Ababa, Debretabor, and South-West Ethiopia revealed similar results, in which women with unintended pregnancy were more than twice as likely to experience depression in their pregnancy [18, 27, 30]. Since pregnancy could bring major changes to the life of the mother, she should be prepared to prevent social, psychological, economical, and health-related problems that could occur due to pregnancy. Our result showed that out of 24 unmarried mothers, 19 of them had unplanned pregnancy. Being pregnant without getting married is unacceptable in Ethiopian culture mothers who are not prepared for the pregnancy may face social stigma which could lead to psychological problems like stress and anxiety. Similarly having a new baby needs financial preparation to maintain the health of the newborn and her.

Primigravida mothers were about three times (OR = 2.96) more likely to develop antenatal depression as compared with multigravida mothers in this study. This result is in line with the study done in Gondar [38]. This could be due to mothers with lack of experience on the physiological and behavioral changes during pregnancy may be fearful and unable to handle new changes.

Mothers who had anxiety in the past two weeks had 5.49times higher odds of developing antenatal depression as compared with mothers without anxiety. This result is in line with the study done in Australia and Malaysia in which mothers with anxiety had 2.15 and 3.17 times higher odds of developing antenatal depression respectively [44, 45]. Mothers with anxiety have uncontrollable worry, difficulty in concentration, disturbed sleep, and impaired occupational and social functioning which might attribute to the occurrence of depression, in which she becomes sad, irritable, and thought suicide [46].

### Limitation of the study

The first limitation of this study was an inability to draw cause-effect relationship among dependent and independent variables and recall bias because the study design used was a cross-sectional study design. The tools used to assess intimate partner violence; social support, anxiety, and threatening life event were not validated in Ethiopia. The sensitivity of the issue related to IPV might have social desirability bias due to the cultural influence in which the mother who experienced violence may report as she hadn’t experienced it. For mothers and their husbands who had no constant monthly income like those who run personal-business, estimate their monthly income was difficult.

## Conclusion and recommendation

The finding of this study revealed that there was a high magnitude of antenatal depressive symptoms, intimate partner violence, and threatening life events in Arba Minch town as compared with other studies done in Ethiopia. The presence of antenatal anxiety, being a primigravida mother, un-intended pregnancy and educational status were the main factors that were significantly associated with antenatal depression in this study.

Though there are women affairs and police offices in the town the offices should work together to create awareness about the adverse effect and illegality of IPV. In addition to this, the ANC clinics should include routine screening of antenatal depression, generalized anxiety, and IPV as a part of focused ANC. Also, there should be a linkage of mothers with IPV to legal authorities. Women should be empowered to tackle the cultural influence of IPV. The governments should work hard to maintain the health of the community and should educate and empower women to have a job and generate income to decrease the prevalence of TLE. In addition to this, policymakers and women affair office must focus on those predictors to reduce antenatal depression.

## Supporting information

S1 File Questionnaire. (DOCX)

## References

[1] Sadock BJ, Sadock VA, Ruiz P. Kaplan & Sadock’s synopsis of psychiatry behavioral sciences/clinical psychiatry. 11th ed. Goolsby J, Elfrank J, Oberle K, Vosburgh A, editors2015.

[2] Marcus M, Yasamy MT, Ommeren Mv, Chisholm D, Saxena S. Depression a global public health concern: W orld Health Organization, Department of Mental Health and Substance Abuse; 2012. Available from: http://www.who.int/mental_health/management/depression/who_paper_depression_wfmh_2012.pdf.

[3] Reynolds CF, Patel V. Screening for depression: the global mental health context. World Psychiatry 2017;16(3):316–7. doi: 10.1002/wps.20459 28941110 PMC5608832

[4] Darkwa EO, Antwi-Boasiako C, Djagbletey R, Owoo C, Obed S, Sottie D. Serum magnesium and calcium in preeclampsia: a comparative study at the Korle-Bu Teaching Hospital, Ghana. Integrated Blood Pressure 2017;10:9–15. 10.2147/IBPC.S129106 PMC5565255 28860856

[5] World Health Organization. Maternal mental health 2016. Available from: http://www.who.int/mental_health/maternal‐child/maternal_mental_health/en/.

[6] Fisher J, Mello MCd, Patel V, Rahman A, Tran T, Holton S, et al. Prevalence and determinants of common perinatal mental disorders in women in lowand lowermiddleincome countries: a systematic review. World Health Organization 2012. 10.2471/BLT.11.091850 PMC3302553 22423165

[7] Pereira PK, Lovisi GM, Lima LA, Legay LF, Santos JFdC, Santos SA, et al. Depression during pregnancy: Review of epidemiological and clinical aspects in developed and developing countries. Uehara T, editor2011.

[8] Getinet W, Amare T, Boru B, Shumet S, Worku W, Azale T. Prevalence and Risk Factors for Antenatal Depression in Ethiopia: Systematic Review. Hindawi Depression Research and Treatment. 2018;2018. doi: 10.1155/2018/3649269 30112199 PMC6077581

[9] Bindt C, Appiah-Poku J, Bonle MT, Schoppen S, Feldt T, Barkmann C, et al. Antepartum depression and anxiety associated with disability in African Women: Cross-sectional results from the CDS study in Ghana and Coˆte d’Ivoire PLOS ONE. 2012;7(10). 10.1371/journal.pone.0048396 PMC3482210 23110236

[10] Brittain K, Myer L, Koen N, Koopowitz S, Donald KA, Barnett W, et al. Risk Factors for Antenatal Depression and Associations with Infant Birth Outcomes: Results From a South African Birth Cohort Study. Paediatric and Perinatal Epidemiology. 2015;29:505–14. doi: 10.1111/ppe.12216 26236987

[11] Tv Heyningen, Myer L, Onah M, Tomlinson M, Field S, Honikman S. Antenatal depression and adversity in urban South Africa. Journal of Affective Disorders. 2016;203:121–9. doi: 10.1016/j.jad.2016.05.052 27285725 PMC5777271

[12] Saeed A, Raana T, Saeed AM, Humayun A. Effect of antenatal depression on maternal dietary intake and neonatal outcome: a prospective cohort Nutr J. 2016;15(64). doi: 10.1186/s12937-016-0184-7 27401187 PMC4939634

[13] Faisal-Cury A, Menezes PR. Antenatal depression strongly predicts postnatal depression in primary health care. Revista Brasileira de Psiquiatria psychatery. 2012;34(4): 446–50. doi: 10.1016/j.rbp.2012.01.003 23429816

[14] NgoI TV, Gammeltoft T, Nguyen HTT, Meyrowitsch DW, Rasch V. Antenatal depressive symptoms and adverse birth outcomes in Hanoi, Vietnam. PloS one. 2018;13(11). 10.1371/journal.pone.0206650 PMC6214542 30388162

[15] Jarde A, Morais M, Kingston D, Giallo R, MacQueen GM, Giglia L, et al. Neonatal Outcome s in Women With Untreated Antenatal Depre ssion Compared With Women Without Depre ssion A Systematic Review and Meta-analysis. JAMA Psychiatry. 2016;73(8):826–37. doi: 10.1001/jamapsychiatry.2016.0934 27276520

[16] Hay DF, Pawlby S, Waters CS, Perra O, Sharp D. Mothers’ Antenatal Depression and Their Children’s Antisocial Outcomes. Child Development. 2010;18(1):149–65. doi: 10.1111/j.1467-8624.2009.01386.x 20331659

[17] Pearson RM, Evans J, Kounali D, Lewis G, Heron J, Ramchandani PG, et al. Maternal depression during pregnancy and the postnatal period: risks and possible mechanisms for offspring depression at age 18 years. JAMA psychiatry. 2013;70(12):1312–9. doi: 10.1001/jamapsychiatry.2013.2163 24108418 PMC3930009

[18] Bisetegn TA, Mihretie G, Muche T. Prevalence and predictors of depression among pregnant women in Debretabor town, Northwest Ethiopia. PLoS ONE. 2016;11(9). doi: 10.1371/journal.pone.0161108 27618181 PMC5019395

[19] Hartley M, Tomlinson M, Greco E, Comulada WS, Stewart J, Mbewu IlRN, et al. Depressed mood in pregnancy: Prevalence and correlates in two Cape Town peri-urban settlements. BMC Journal of reproductive Health. 2011;8(9). doi: 10.1186/1742-4755-8-9 21535876 PMC3113332

[20] Zeng Y, Cui Y, Li J. Prevalence and predictors of antenatal depressive symptoms among Chinese women in their third trimester: a cross sectional survey. BMC Psychiatry. 2015;15(66). doi: 10.1186/s12888-015-0452-7 25879965 PMC4387591

[21] Tesfaye M, Hanlon C, Wondemagegn D, Alem A. Detecting postnatal common mental disorders in Addis Ababa, Ethiopia: validation of Edinbergh postnatal depression scale and Kessler scale. Journal of Affective Disorders. 2010;122(1–2):102–8. doi: 10.1016/j.jad.2009.06.020 19615753

[22] Hanlon C, Medhin G, Alem A, Araya M, Abdulahi A, Hughes M, et al. Detecting perinatal common mental disorders in Ethiopia: validation of the self-reporting questionnaire and Edinburgh Postnatal Depression. Journal of Affective Disorders. 2008; 108(3):251–62. doi: 10.1016/j.jad.2007.10.023 18055019

[23] Gemta WA. prevalence and factors associated with antenatal depression among women following antenatal care at Shasemane health facilities, south Ethiopia. journal of annals of global health. 2015;81(1). doi: 10.1016/j.aogh.2015.01.004 25770696

[24] Rubertsson C, Borjesson K, Berglund A, Josefsson A, Sydsjo G. the swedish validation of edinburgh postpartum depression scale (EPDS) during pregnancy. Nordic Journal of Psychiatry. 2011;65(6):414–8. doi: 10.3109/08039488.2011.590606 21728782

[25] Adewuya A, Ola B, Dada A, Fasoto O. Validation of the Edinburgh Postnatal Depression Scale as a screening tool for depression in late pregnancy among Nigerian women. J Psychosom Obstet Gynaecol. 2006;27(4):267–72. doi: 10.1080/01674820600915478 17225628

[26] Gibson J, Mckenzie-McHarg K, Shakespeare J, Prince J, Gray R. Asystematic review of stidies validating edinuburgh postnatal depression scale in antepartum and postpartum women. Acta Psychiatr Scand. 2009;119(5):350–64. doi: 10.1111/j.1600-0447.2009.01363.x 19298573

[27] Biratu A, Haile D. Prevalence of antenatal depression and associated factors among pregnant women in Addis Ababa, Ethiopia: a cross-sectional study. Reproductive health. 2015;12:99. doi: 10.1186/s12978-015-0092-x 26514827 PMC4627391

[28] Dadi AF, Dachew BA, Kisi T, Yigzaw N, Azale T. Anxiety and associated factors among prisoners in North West of Amhara Regional State, Ethiopia. BMC Psychiatry. 2016;16(83). doi: 10.1186/s12888-016-0792-y 27036945 PMC4815165

[29] Zhong Q-Y, Gelaye B, Zaslavsky AM, Fann JR, Rondon Marta B., Sánchez SE, et al. Diagnostic Validity of the Generalized Anxiety Disorder—7 (GAD-7) among Pregnant Women. PLoS ONE. 2015;10(4). doi: 10.1371/journal.pone.0125096 25915929 PMC4411061

[30] Dibaba Y, Fantahun M, Hindin MJ. The association of unwanted pregnancy and social support with depressive symptoms in pregnancy: evidence from rural Southwestern Ethiopia. BMC Pregnancy and Childbirth. 2013;13(135). doi: 10.1186/1471-2393-13-135 23800160 PMC3716614

[31] Rabin RF, MeganH.Bair. Intimate partner violence screening tools. American journal of preventive medicine. 2009;36(5):439–45. doi: 10.1016/j.amepre.2009.01.024 19362697 PMC2688958

[32] Weiss SJ, Ernst AA, Cham E, Nick TG. Development of a screen for ongoing intimate partner violence. Violence and Victims. 2003;18(2):131–41. doi: 10.1891/vivi.2003.18.2.131 12816400

[33] Reichenheim ME, Moraes CL. Comparison between the abuse assessment screen and the revised conflict tactics scales for measuring physical violence during pregnancy. J Epidemiol Commun Health Care. 2004;58(6):523–7. doi: 10.1136/jech.2003.011742 15143123 PMC1732799

[34] Gerberding JL, Falk H, Arias I, Hammond WR. Intimate partner violence and sexual violence victimization assessment instruments for use in health care settings. In: Prevention CfDCa, editor. Atlanta (GA): National center for injury prevention and control; 2007.

[35] Montón-Franco C, Josefa G, Gómez-Barragán M, Sánchez-Celaya M, ÁngelDíaz-Barreiros M. Psychometric properties of the List of Threatening Experiences—LTE and its association with psychosocial factors and mental disorders according to different scoring methods. JAffectDisord. 2013;150:931–40. 10.1016/j.jad.2013.05.017 23726778

[36] Shruthi H, Keshava KP, Hulegar AA, Sandeep KR. Prevalence of antenatal depression and gender preference: A cross sectional study among mangalore population,Karnataka,India. J Pharm Biomed Sci. 2013;30:1011–4.

[37] Rwakarema M, Premji SS, Nyanza EC, Riziki P, Palacios-Derflingher L. Antenatal depression is associated with pregnancy-related anxiety, partner relations, and wealth in women in Northern Tanzania: a cross-sectional study. BMC Women’s Health. 2015;15(68). 10.1186/s12905-015-0225-y PMC4557331 26329331

[38] Ayele TA, Azale T, Alemu K, Abdissa Z, Mulat H, Fekadu A. Prevalence and associated factors of antenatal depression among women attending antenatal care service at Gondar University Hospital, Northwest Ethiopia. PLoS ONE. 2016;11(5). doi: 10.1371/journal.pone.0155125 27153193 PMC4859574

[39] Trott CD, Harman JJ, Kaufman MR. Women’s Attitudes Toward Intimate Partner Violence in Ethiopia: The Role of Social Norms in the Interview Context. Violence Against Women. 2017;23(8): 1016–36. doi: 10.1177/1077801216654018 27364004

[40] Alebel A, Kibret GD, Wagnew F, Tesema C, Ferede A, Petrucka P, et al. Intimate partner violence and associated factors among pregnant women in Ethiopia: a systematic review and metaanalysis. BMC Journal of reproductive Health. 2018;15(196). 10.1186/s12978-018-0637-x PMC6278116 30514311

[41] Stewart RC, Umar E, Tomenson B, Creed F. A cross-sectional study of antenatal depression and associated factors in Malawi. Arch Womens Ment Health 2014;17: 145–54. doi: 10.1007/s00737-013-0387-2 24240635

[42] Thompson O, Ajayi I. Prevalence of antenatal depression and associated risk factors among pregnant women attending antenatal clinics in Abeokuta north local government area, Nigeria. depression research and treatment [Internet]. 2016. Available from: doi: 10.1155/2016/4518979 27635258 PMC5007324

[43] Al-Azri M, Al-Lawati I, Al-Kamyani R, Al-Kiyumi M, Al-Rawahi A, Davidson R, et al. Prevalence and risk factors of antenatal depression among Omani women in a primary care setting Cross-sectional study. Sultan Qaboos University Med J. 2016;16(1):e35–41. 10.18295/squmj.2016.16.01.007 PMC4746041 26909211

[44] Yusuff ASM, Tang L, Binns CW, Lee AH. Prevalence of antenatal depressive symptoms among women in Sabah, Malaysia. J Matern Fetal Neonatal Med. 2015. 10.3109/14767058.2015.1039506 26037724

[45] Leigh B, Milgrom J. Risk factors for antenatal depression, postnatal depression and parenting stress. BMC Psychiatry. 2008;8(24). 10.1186/1471-244X-8-24 PMC2375874 18412979

[46] Hoge EA, Ivkovic A, Fricchoine GL. Generalized anxiety disorder: diagnosis and treatment. BMJ. 2012;345. doi: 10.1136/bmj.e7500 23187094

