# Supplementary material for: Magnitude and associated factors of antenatal depression among mothers attending antenatal care in Arba Minch town, Ethiopia, 2018
Source: PLoS One. 2021 Dec 2;16(12):e0260691. doi: 10.1371/journal.pone.0260691 (PMC8638914; doi:10.1371/journal.pone.0260691)
Supplement: S1 File — (DOCX) [file pone.0260691.s001.docx]

English version questionnaire

Hello. My name is _____________________I am collecting data for research titled “Magnitude and associated factors of antenatal depression anong pregnant women attending Public Health facilities in Arba Minch town SNNPR Ethiopia 2018”. We received permission from Mekelle University College of health science to conduct this study. You have been selected to participate in this study as you attend ANC in one of selected health institution.

The aim of the study is to assess the magnitude and factors associated with antenatal depression. If you are willing to participate I will ask you some questions concerning your socio-demographic, health status, and reproductive history. The interview will last no more than 15 minutes. Your participation in the study is voluntary. You may ask any questions during the interview, or skip any question you think is inappropriate and stop it at any moment you want with no further negative consequences.

The information collected from you will be kept confidential. It will be stored in a file using codes, without your name. And it will be exposed to no one except the investigators. In addition it will be used only for this particular research but no other purposes. Your participation in the study poses no risk for you.

I comprehend and understood the condition stated above, therefore, I am willing and confirm my participation by signing the consent.

Agreed to participate in the study: Yes □ No□

ID _______________

| 1. **Demographic and Socioeconomic factors** | | |
| --- | --- | --- |
| Serial No | Question | Response |
|  | How old are you? | __________________ |
|  | What is your ethnicity? | 1. Gamo Gofa 2. Wolayta 3. Amara 4. Oromo 5. Others (specify)________ |
|  | What is your religion? | 1. Orthodox tewahido 2. Protestant 3. Muslim 4. Other specify____________ |
|  | Did you had regular education? | 1. Yes 2. No |
|  | If the answer for question number 104 is yes what is your educational status? | ______________________ |
|  | What is your occupation | 1. Government employee 2. Private employee 3. Running personal business 4. House wife 5. Student 6. Jobless |
|  | Marital status | 1. Never married 2. Married or living together 3. Divorced 4. Separated 5. Widowed |
|  | Estimated monthly income of any source | ____________________ |

| 1. **Edinburgh postpartum depression scale. Have you faced the these problems in the past 7 days** | | |
| --- | --- | --- |
| Serial No | Question | Response |
| 201 | I have been able to laugh and see the funny side of things | 1. As much as I always could 2. Not quite so much now 3. Definitely not so much now 4. Not at all |
|  | I have looked forward with enjoyment to things | 1. As much as I ever did 2. Rather less than I used to 3. Definitely less than I used to 4. Hardly at all |
|  | I have blamed myself unnecessarily when things went wrong | 1. Yes, most of the time 2. Yes, some of the time 3. Not very often 4. No, never |
|  | I have been anxious or worried for no good reason | 1. No, not at all 2. Hardly ever 3. Yes, sometimes 4. Yes, very often |
|  | I have felt scared or panicky for no very good reason | 1. Yes, quite a lot 2. Yes, sometimes 3. No, not much 4. No, not at all |
|  | Things have been getting on top of me | 1. Yes, most of the time I haven’t been able to cope at all 2. Yes, sometimes I haven’t been coping as well as usual 3. No, most of the time I have coped quite well 4. No, have been coping as well as ever |
|  | I have been so unhappy that I have had difficulty sleeping | 1. Yes, most of the time 2. Yes, sometimes 3. Not very often 4. No, not at all |
|  | I have felt sad or miserable | 1. Yes, most of the time 2. Yes, quite often 3. Not very often 4. No, not at all |
|  | I have been so unhappy that I have been crying | 1. Yes, most of the time 2. Yes, quite often 3. Only occasionally 4. No, never |
|  | The thought of harming myself has occurred to me | 1. Yes, quite often 2. Sometimes 3. Hardly ever 4. Never |

| 1. **Obstetric gynecologic factors** | | |
| --- | --- | --- |
| Serial No | Question | Response |
|  | Have you ever had irregular menstrual cycle? | 1. Yes 2. No |
|  | Before this pregnancy did you used contraceptive method? | 1. Yes 2. No |
|  | What kind of contraceptive you have been using before this pregnancy? | 1.OCP   1. Dipo 2. Implant 3. IUCD |
|  | Is current pregnancy planned? | 1. Yes 2. No |
|  | How many times you have been pregnant? | ______________ |
|  | How many times you give birth to a baby (still birth & live birth)? | ________________ |
|  | How many children do you have? | __________________ |
|  | Have you ever faced complication in the previous pregnancy or labor? | 1. Yes 2. No |
|  | If the answer for question no. 208 is yes what type of complication? | 1. Abortion 2. Preterm birth 3. Stillbirth 4. Others __________ |
|  | If you faced abortion in the previous pregnancies what type of abortion was it? | 1. Spontaneous 2. Induced |
|  | Is there any complication that you are facing in the current pregnancy? | 1. Yes 2. No |
|  | In which trimester you are in? | ______________ |
|  | Did you had ANC follow up in the previous pregnancies? | - 1. Yes   2. No |
|  | If the answer for question number 212 is yes how did you follow ANC. | 1. Irregularly 2. Regularly |

| 1. **Psychosocial factors** | | | |
| --- | --- | --- | --- |
| Serial No | Question | Response | |
|  | What is your Partner’s feeling on current pregnancy | 1. Happy 2. Un-happy | |
|  | How is baby’s fathers support | 1. Poor 2. Good | |
|  | **Lists of threatening life events**  Have you ever faced the following life events in the last six months? | | |
|  | Serious illness or injury to you? | | 1. Yes 2. No |
|  | Serious illness or injury to your close relative? | | 1. Yes 2. No |
|  | Death of first degree relative including child or spouse? | | 1. Yes 2. No |
|  | Death of close family, friend or second degree relative? | | 1. Yes 2. No |
|  | Separation due to marital difficulties? | | 1. Yes 2. No |
|  | Broke off a steady relationship? | | 1. Yes 2. No |
|  | Serious problem with close neighbor, friend or relative? | | 1. Yes 2. No |
|  | Un-employed/ seeking work for more than one month? | | 1. Yes 2. No |
|  | You sacked from job? | | 1. Yes 2. No |
|  | Do you have major financial crisis? | | 1. Yes 2. No |
|  | Problem with police and court appearance? | | 1. Yes 2. No |
|  | Something valuable lost or stolen? | | 1. Yes 2. No |
|  | **Abuse Assessment Screen** | | |
|  | Have you ever been emotionally or physically abused by your partner? | | - 1. Yes   2. No |
|  | Within the last year, have you been hit, slapped, kicked or otherwise physically hurt by your partner? | | 1. Yes 2. No |
|  | Since you’ve been pregnant, have you been slapped, kicked or otherwise physically hurt by your partner? | | 1. Yes 2. No |
|  | Within the last year, has your partner forced you to have sexual activities? | | 1. Yes 2. No |
|  | Are you afraid of your partner? | | 1. Yes 2. No |

| **Maternity social support scale** | | | | | | |
| --- | --- | --- | --- | --- | --- | --- |
| Serial No | Question | Response | | | | |
|  |  | Always | Most of the time | Some of the time | Rarely | Never |
| 405 | I have good friends who support me | 5 | 4 | 3 | 2 | 1 |
|  | My family is always there for me | 5 | 4 | 3 | 2 | 1 |
|  | My husband/ partner helps me a lot | 5 | 4 | 3 | 2 | 1 |
|  | There is conflict with my husband or partner | 1 | 2 | 3 | 4 | 5 |
|  | I feel controlled by my husband/ partner | 1 | 2 | 3 | 4 | 5 |
|  | I feel loved by my husband/ partner | 5 | 4 | 3 | 2 | 1 |

| 1. **Mental health conditions** | | |
| --- | --- | --- |
| Serial No | Question | Response |
| 501 | Do you have history of depression? (Have you ever been diagnosed with depression?) | 1. Yes 2. No |
| 502 | Did anyone from your family had depression history?  (Anyone from your family ever been diagnosed with depression?) | 1. Yes 2. No |

| **Generalized Anxiety Disorder 7-Item Scale** | | | | | |
| --- | --- | --- | --- | --- | --- |
| Serial No | Question | Response | | | |
|  |  | Not at  all sure | Several  days | Over half  the days | Nearly  every day |
| 503 | Over the last 2 weeks, how often have you been bothered by the following problems? | | | | |
|  | Feeling nervous, anxious, or on edge | 0 | 1 | 2 | 3 |
|  | Not being able to stop or control worrying | 0 | 1 | 2 | 3 |
|  | Worrying too much about different things | 0 | 1 | 2 | 3 |
|  | Trouble relaxing | 0 | 1 | 2 | 3 |
|  | Being so restless that it's hard to sit still | 0 | 1 | 2 | 3 |
|  | Becoming easily annoyed or irritable | 0 | 1 | 2 | 3 |
|  | Feeling afraid as if something awful might happen | 0 | 1 | 2 | 3 |

| 1. **Substance use** | | |
| --- | --- | --- |
| Serial No | Question | Response |
| 601 | Did you drink alcohol in this pregnancy? | 1. Yes 2. No |
|  | Do you smoke cigarette in this pregnancy? | 1. Yes 2. No |
|  | Do you chew chat in this pregnancy? | 1. Yes 2. No |

Amharic version questioner

በአርባ ምንጭ በሚገኙ የመንግስት የጤና ተቋማት ውስጥ የቅድመ ወሊድ ክትትል ለማድረግ የመጡ እናቶች ላይ በእርግዝና ጊዜ የሚከሰትን ድብርትና ተያየዥ ምክንያቶችን ለመዳሰስ የተዘጋጀ መጠይቅ

መግብያ

ጤና ይስጥልኝ ስሜ_____________________ይባላል፡፡ የመጣሁት በእርግዝና ጊዜ የሚከሰት ድብርትና ተያያዥ ምክንያቶችን በተመለከተ መረጃ ለመስብብ ነው፡፡

ይህን ለመስራት ደግሞ በመቐለ ዩኒቨርስቲ ጤና ሳይንስ ኮሌጅ የነርሲንግ ትምህርት ቤት ፈቃድ አለኝ፡፡ መረጃው የሚሰበሰበው በአርባምንጭ በሚገኙ የመንግስት የጤና ተቋማት ነው፡፡ በጤና ተቋማቱ የቅድመ ወሊድ ክትትል ለማድረግ በመምጣትዎ በእጣ ጥናቱ ላይ ተሳታፊ ሆኖዋል፡፡ ይህን መረጃ ለመሰብሰብ የጤና ተቋሙን ሀላፊ ያስፈቀድን ሲሆን እርሰዎ የሚሰጡን መረጃ ሚስጥራዊነቱ የተጠበቀ እና ለጥናቱ ብቻ የሚውል ነው፡፡ ስለዚህ ፈቃደኛ ከሆኑ አንድ አንድ ከጤና ጋር የተያያዙ መረጃዎችን ከ15 ደቂቃ ያልበለጠ እጠይቅዎታለሁ፡፡

ጥናቱ ላይ የሚሳተፉት በፈቃድኝነት ስለሆነስጠይቅዎት በመሀል የመጠየቅ፤ ጥያቄውን የመዝለል፤ ብሎም የማስቆም መብት አለዎት የእርሰዎ ጥናቱ ላይ መሳተፍ አሁን ለግልዎ ጥቅም ባይኖረውም የሚሰጡኝ መረጃ ግን በእርግዝና ወቅት የሚከሠትን ድብርትና ተያያዥ ምክንያቶች ለመዳሰስ ትልቅ ጥቅም አለው፡፡ እርሰዎ ጥናቱ ላይ በመሳተፎዎ የሚደርስብዎት ችግር የለም፡፡

እስካሁን ከነገርኩዎት ወይም ጥናቱ በመለከተ ሊብራራልዎት የሚፈልጉት ነገር አለ?

አሁን ጥናቱ ላይ ለመሳተፍ ፈቃደኛ ነዎት? አዎ □ አይደለሁም □

ጥናቱ ላይ ስለተሳተፉ አመሰግናለሁ፡፡

የስምምነት ቅጽ

በእርግዝና ጊዜ የሚከሰትን ድብርትና ተያያዥ ምክንያቶችን ለመዳሰስ በሚደረገው ጥናት ለመሳተፍ ፈቃደኛ ነኝ፡፡ ስለማህበራዊ ኩነቶች፤ እርግዝናና ወሊድ ኩነቶች፤ ማህበራዊ ሕይወትን የሚመለከቱ ኩነቶች፤ ስነአዕምሮ ጤና ኩነቶች እና ዕፅ ተጣቃሚነት ጋር የተያያዙ ጥያቄዎች እንደምጠየቅ ተገንዝቤአለሁ፡፡ ጥናቱ በአርባ ምንጭ በሚገኙ የመንግስት የጤና ተቋማት ውስጥ የሚደረግ ሲሆን 15 ደቂቃ እንደሚወስድብኝ ተነግሮኛል፡፡ በተጨማሪም ጥያቄ መዝለል ብሎም ማስቆም እንደምችል የተነገረኝ ሆኖ መረጃው ለጥናቱ ብቻ የሚውል ከመሆኑ ጋር ተያይዞ በጥናቱ ላይ የምሳተፈው በፍላጎቴ መሆኑን በፊርማዬ አረጋግጣለሁ፡፡

የተጠያቂው ፊርማ --------------------------ቀን--------------------------

የጠያቂው ፊርማ-----------------------------ቀን-----------------------

መ.ቁ ______________________

| 1. ስነ ህዝብና ማህበራዊ ኩነቶች ጋር የተያየዙ ጥያቄዎች | | |
| --- | --- | --- |
| ተ.ቁ | ጥያቄዎች | አማራጭ መልሶች |
|  | ዕድሜሽ ስንት ነው? | _____________ |
|  | ብሄርሽ ምንድን ነው? | 1. ጋሞ 2. ወላይታ 3. አማራ 4. ኦሮሞ 5. ሌላካለይጠቀስ |
|  | ሀይማኖትሽ ምንድን ነው ? | 1. ኦርቶዶክርስ 2. ፕሮቴስታንት 3. ሙስሊም 4. ሌላካለይጠቀስ____________ |
|  | መደበኛ ትምህርት ተምረሽ ታውቂያለሽ? | - 1. አዎ   2. አልተማርኩም |
|  | ለጥያቄ ቁጥር 104 መልስሽ አዎ ከሆነ የትምህርት ደረጃሽ ስንት ነው? (ያጠናቀቅሽው) | _________________ |
|  | የስራ ድርሻሽ ምንድን ነው? | 1. የመንግስት ሰራተኛ 2. የግልተቀጣሪ 3. ነጋዴ 4. የቤት እመቤት 5. ተማሪ 6. ስራአጥ |
|  | የትዳርሽ ሁኔታ | 1. አላገባሁም 2. አግብቻለሁ/አብረን እንኖራለን 3. ተፋተናል 4. ተለያይተን ነው የምንኖረው 5. ባለቤቴ በሕይወት የለም |
|  | በአማካይ ግምታዊ የቤተሰብሽ ገቢ በወር ስንት ይሆናል? | _____________________ |

| 2. ድብርትን የሚመለከቱ ጥያቄዎች  በአለፉት 7 ቀናት የተሰማሽ ስሜት ካለ ከዚያ ስሜት ጋር የሚጠጋውን አማራጭ ምረጪ | | |
| --- | --- | --- |
| ተ.ቁ | ጥያቄዎች | አማራጭ መልሶች |
|  | ትስቂአለሽ? አስቂኝ ሁኔታዎችንም መለየት ችለሻል? | 1. በፊት ስታደርጊው እንደነበረው 2. እንደ በፊቱ አይሆንም 3. በጭራሽ እንደበፊቱ አይሆንም 4. በጭራሽ አትስቂም |
|  | መጪውን ሁኔታ በደስታ መቀበል ትችያለሽ? | 1. በፊት የምታደርጊውን ያህል 2. በፊት ከምታደርጊው አነስ ያለ 3. በእርግጥ በፊት ከምታደርጊውያነስ 4. በጭራሽ አትችይም |
|  | ሁኔታዎች ሳይሳኩ ከቀሩ አራስሽን እወቅሳለሁ? | 1. አዎን፤ ሁልጊዜ 2. አዎን፤ አልፎ አልፎ 3. እስከዚህም እራስሽን አትወቅሽም 4. በጭራሽ እራስሽን አትወቅሽም |
|  | በማይረባ ነገር ትጨነቂያለሽ ፤ ትጠበቢያለሽ? | 1. በጭራሽ 2. ከቁጥር ለማይገባ ጊዜ 3. አዎን፤ አልፎ አልፎ 4. አዎን፤ ሁልጊዜ |
|  | በማይረባ ምክንያት ፍርሃትና ድንጋጤ ይሰማሻል? | 1. አዎን፤ ሁልጊዜ 2. አዎን፤ አልፎአልፎ 3. እስከዚህ አይሰማሽም 4. በጭራሽ አይሰማሽም |
|  | ሁኔታዎች ተደራርበውብሻል? | 1. አዎን፤ አብዛኛውን ጊዜ ሁኔታዎችን መቋቋም አትችይም 2. አዎን፤ አልፎ አልፎ ሁኔታዎችን መቋቋም አትችይም 3. አይ፤ አብዛኛውን ጊዜ ሁኔታዎችን መቋቋም ትችያለሽ 4. አይ፤ በፊት እንደምታደርጊው ሁኔታዎችን መቋቋም ትችያለሽ |
|  | ደስታ ከማጣትሽ የተነሳ እንቅልፍ አይወስድሽም? | 1. አዎን፤ ሁልጊዜ 2. እዎን፤ አልፎ አልፎ 3. እስከዚህም አልተቸገርሽም 4. በጭራሽ አልተቸገርሽም |
|  | ብስጭትና ሃዘን ተሰምቶሻል? | 1. አዎን፤ ሁልጊዜ 2. አዎን፤ አልፎ አልፎ 3. እስከዚህ አይሰማሽም 4. በጭራሽ አይሰማሽም |
|  | ደስታ ከማጣትሽ የተነሳ ታለቅሻለሽ? | 1. አዎን፤ አብዛኛውን ጊዜ 2. አዎን፤ በየጊዜው 3. አልፎ አልፎ ብቻ 4. በጭራሽ አላለቀስሽም |
|  | እራስሽን የመጉዳት ሃሳብ ደርሶብሻል? | 1. አዎን፤ ሁልጊዜ 2. አልፎ አልፎ ገጥሞሻል 3. እስከዚህም አልገጠመኝሽም 4. በጭራሽ አልገጠመሽም |

| 1. እርግዝና እና ወሊድን የሚመለክቱ ጥያቄዎች | | |
| --- | --- | --- |
| ተ.ቁ | ጥያቄዎች | አማራጭ መልሶች |
|  | ከእርግዝናሽ በፊት የወር አበባ መዛባት አጋጥሞሽ ያውቃል? | 1. አዎ 2. አያዉቅም |
|  | ከእርግዝናሽ በፊት የወሊድ መከላከያ ትጠቀሚ ነበር? | 1. አዎ 2. አልጠቀምም |
|  | ለጥያቄ ቁጥር 202 መልሱ አዎ ከሆነ ምን አይነት የእርግዝና መከላከያ ዘዴ ነበር? | 1 የሚዋጥ እንክብል  2 በመርፌ የሚሰጥ  3 በክንድ የሚቀበር  4 በማህፀን የሚቀመጥ |
|  | የአሁኑ እርግዝናሽ ታቅዶበት ነው የተረገዘው? | 1. አዎ 2. አይደለም |
|  | ከዚህ በፊት ምን ያህል ጊዜ አርግዘሽ ያውቂያለሽ? | ______________ |
|  | ከዚህ በፊት ምን ያህል ልጆች ወልደሻል? | ________________ |
|  | አሁን ቤት ስንት ልጆች አሉሽ? | __________________ |
|  | ከዚህ በፊት በነበረሽ እርግዝና የጤና እክል አጋጥሞሽ ነበር?(ከአንድ ጊዜ በላይ ላረገዙ እናቶች) | 1. አዎ 2. አላጋጠመሽም |
|  | ለጥያቄ ቁጥር 208 የሠጡት ምላሽ አዎ ከሆነ ምን አይነት የጤና እክል ነበር ያጋጠመሽ? | 1. የፅንስ መቋረጥ 2. ከቀኑ ቀድሞ መወለድ 3. በሕይወት ያላተወለደ 4. ለላካለ ይጠቀስ_______ |
|  | ለጥያቄ ቁጥር 209 የሰጠሸው ምላሽ የፅንስ መቋረጥ ከሆነምን አይነት የፅንስ መቋረጥ ነበር? | 1. ፅንሱ የተቋረጠዉ በራሱ ነበር 2. የተለያዩ ዘዴዎችን ተጠቅመሽ ነው ፅንሱ የተቋረጠው |
|  | በአሀኑ እርግዝናሽ ያጋጠመሽ የጤና እክል አለ?(ከካርድ ይመልከቱ) | 1. አዎ 2. የለም |
|  | ያአሁኑ እርግዝናሽ ስንተኛ ሳምንቱ ነው? (ከካርድ ይመልከቱ) | ________________________ |
|  | ከዚህ በፊት ለነበረሽ እርግዝና የቅድመ ወሊድ ክትትል ነበረሽ? | - 1. አዎ   2. አልነበረሽም |
|  | ለጥያቄ ቁጥር 213 መልስሽ አዎ ከሆነ ክትትል ታደርጊ የነበረዉ እንዴት ነበር.? | 1. አልፎ አልፎ ትከታተይ ነበር 2. በትክክል በቀጠሮ ትከታተይ ነበር |

| 1. ማህበራዊ ህይወትን የሚመለከቱ ጥያቄዎች | | | |
| --- | --- | --- | --- |
| ተ.ቁ | ጥያቄዎች | | አማራጭ መልሶች |
|  | ባለቤትሽ/የልጅሽ አባት በአሁኑ እርግዝናሽ ምን አይነት ስሜት አለው? | | 1. ደስተኛ ነዉ 2. ደስተኛ አይደለም |
|  | ባለቤትሽ/የልጅሽ አባት የሚያደርግልሽ ድጋፍ ምን ይመስላል? | | 1. ጥሩ አይደለም 2. ጥሩነው |
|  | የህይወት አጋጣሚዎችን የሚመለከቱ ጥያቄዎች  ባለፉት ስድስት ወራት ውስጥ የሚተሉት የህይወት አጋጣሚዎች አጋጥሞሻል? | | |
|  | ከፍተኛ የጤና እክል ወይም አደጋ በራስሽ ላይ ገጥሞሽ ያውቃል? | 1. አዎ 2. አልገጠመኝም | |
|  | ከፍተኛ የጤና እክል ወይም አደጋ ለቅርብ ዘመድሽ ገጥሞ ያውቃል? | 1. አዎ 2. አልገጥመኝም | |
|  | የቅርብ ዘመድሽ፤ ባለቤትሽ ወይም ልጆችሽ በሕይወት ተለይተውሻል ? | 1. አዎ 2. አልተለዩኝም | |
|  | ጓዳኛ ወይም የሩቅ ዘመድ በህይወት ተለይተውሻል? | 1. አዎ 2. አልተለዩቶኝም | |
|  | በትዳር ባለመስማማት ከባለቤትሽ ጋር ተለያይተሻል? | 1. አዎ 2. አልተለየሁም | |
|  | ሰላማዊ የነበረ ግንኙነት (ጉርብትና) አቋርጠሻል ? | 1. አዎ 2. አላቋረጥኩም | |
|  | ከቅርብ ጎረቤትዎ፤ ከጓዳኛዎ ወይም ከዘመድዎ ጋር የከራራ ግጭት አለሽ? | 1. አዎ 2. የለም | |
|  | ስራ አጥ ነሽ /ከአንድ ወር በላይ ስራ ስትፈልጊ ነበር ? | 1. አዎ 2. አይደለሁም | |
|  | የስራ ቦታሽ ሰልችቶሻል? | 1. አዎ 2. አልሰላቸኝም | |
|  | ከበድ ያለ የኢኮኖሚ መቃወስ አጋጥሞሻል? | 1. አዎ 2. አላጋጠመኝም | |
|  | ከፖሊስ ጋር ወይም ፍርድ ቤት የሚያስኬድዎ ጉዳይ ነበረሽ ? | 1. አዎ 2. አልነበረኝም | |
|  | ዋጋ የሚያወጣ ንብረት ጠፍቶብሻል ወይም ተሰርቆብሻል ? | 1. አዎ 2. አልጠፋብኝም /አልተሰረቀብኝም | |
| 404 | የአዕምሮ ጭቆናን የሚመለከቱ ጥያቄዎች | | |
|  | በባለቤትሽ የአካል ወይም የአዕምሮ ጭቆና አድርሶብሽ ያውቃል? | - 1. አዎ   2. አያዉቅም | |
|  | ባለፈዉ 1 አመት ውስጥ በባለቤትሽ ተመተሽ፤ ተደድበሽ ወይም የአካል ጉዳት ደርሶብሽ ያዉቃል? | - 1. አዎ   2. አያዉቅም | |
|  | እርጉዝ ከሆሽ ጀምሮ በባለቤትሽ ተመተሸ፤ ተደድበሽ ወይም የአካል ጉዳት ደርሶብሽ ያዉቃል? | 1. አዎ 2. አያዉቅም | |
|  | ባለፈዉ 1 አመት ውስጥ ባለቤትሽ ያለፈቃድሽ ፆታዊ ግንኙነት እንድታደርጊ አስገድዶሽ ያዉቃል? | 1. አዎ 2. አያዉቅም | |
|  | ባለቤትሽን ትፈሪዋለሽ? | 1. አዎ 2. አልፈራዉም | |

ማህበራዊ ድጋፍን የሚመለከቱ ጥያቄዎች

| ተ.ቁ | ጥያቄዎች | አማራጭ መልሶች | | | | |
| --- | --- | --- | --- | --- | --- | --- |
|  |  | ሁልቀን | እስማማለሁ | ገልተኛነኝ | አልስማማም | በጣምአልስማማም |
| 405 | የሚረዱሽ/የሚደግፉሽ ጥሩ ጓዳኛዎች አሉሽ? | 5 | 4 | 3 | 2 | 1 |
|  | ቤተሰቦችሽ ሁል ጊዜ ከጎንሽ ናቸው? /ይደግፉሻል? | 5 | 4 | 3 | 2 | 1 |
|  | ባለቤትሽ በጣም ያግዝሻል/ ይረዳሻል? | 5 | 4 | 3 | 2 | 1 |
|  | ከባለቤትሽ ጋር አለመስማማት/ ግጭት አለሽ? | 1 | 2 | 3 | 4 | 5 |
|  | ባለቤትሽ የሚቆጣጠርሽ አይነት ስሜት ይሰማሻል? | 1 | 2 | 3 | 4 | 5 |
|  | በባለቤትሽ የተወደድሽ አይነት ስሜት ይሰማሻል? | 5 | 4 | 3 | 2 | 1 |

| 5. የአዕምሮ ጤንነትን የሚመለከቱ ጥያቄዎች | | |
| --- | --- | --- |
| ተ.ቁ | ጥያቄዎች | አማራጭ መልሶች |
| 501 | ከዚህ በፊት በድብርት ህመም ታመሽ ታዉቂያለሽ ? (በድብርት ምክንያት ሀኪም ቤት ሄደሽ ታክመሽ ታዉቂያለሽ?) | 1. አዎ 2. አላዉቅም |
| 502 | ከቤተሰብሽ መካከል በድብርት ህመም የታመመ/ ታሞ የሚያውቅ/ ሰው አለ?(በድብርት ምክንያት ሀኪም ቤት ሄዶ የታክመ ቤተሰብ አለሽ?) | 1. አዎ 2. የለም |

| ስጋትን የሚመለከቱ ጥያቄዎች | | | | | |
| --- | --- | --- | --- | --- | --- |
| ተ.ቁ | ጥያቄዎች | አማራጭ መልሶች | | | |
|  |  | በፍፁም | አንድአንድቀን | አብዘኛዉንቀን | ሁልቀን |
| 503 | ላለፉት ሁለት ሣምንታት በሚከተሉት ችግሮች ምን ያህል ተቸግረሻል? | | | | |
|  | የመደናገጥ የመረበሽ ስሜት አለሽ? | 0 | 1 | 2 | 3 |
|  | መጨነቅ ማቆም አልቻልሽም? | 0 | 1 | 2 | 3 |
|  | ስለ ተለያዩ ነገሮች ከሚገባው /ከመጠን/ በላይ ትጨነቂያለሽ? | 0 | 1 | 2 | 3 |
|  | ዘና የማለት ችግር አጋጥሞሻል? | 0 | 1 | 2 | 3 |
|  | መረጋጋት አልቻልሽም/ አንድ ቦታ ረግቶ መቀመጥ አቅቶሻል? | 0 | 1 | 2 | 3 |
|  | በቀላሉ ትናደጃለሽ ወይም ትበሳጫለሽ? | 0 | 1 | 2 | 3 |
|  | መጥፎ ነገር ይፈጠራል ብለሽ ትፈሪያለሽ? | 0 | 1 | 2 | 3 |

| 6. ዕፅ መጠቀምን የሚመለከቱ ጥያቄዎች | | |
| --- | --- | --- |
| ተ.ቁ | ጥያቄዎች | አማራጭ መልሶች |
| 601 | በዚህ እርግዝናሽ የአልኮል መጠጥ ጠጥተሽ ታዉቂያለሽ? | 1. አዎ 2. አላዉቅም |
| 602 | በዚህ እርግዝናሽ ሲጋራ አጭሰሽ ታዉቂያለሽ? | 1. አዎ 2. አላዉቅም |
| 603 | በዚህ እርግዝናሽ ጫት ቅመሽ ታዉቂያለሽ? | 1. አዎ 2. አላዉቅም |
